# New Frontiers in Antegrade Wiring From the Asia Pacific Chronic Total Occlusion Club

**DOI:** 10.1016/j.jacasi.2024.12.009

**Published:** 2025-02-04

**Authors:** Eugene B. Wu, Shunsuke Matsuno, Wataru Nagamatsu, Arun Kalyanasundaram, Scott A. Harding, Sidney Lo, Soo Teik Lim, Lei Ge, Ji-Yan Chen, Henry J.F. Luo, Jie Quan, Seung-Whan Lee, Hsien-Li Kao, Etsuo Tsuchikane

**Affiliations:** a Prince of Wales Hospital, Hong Kong; b The Cardiovascular Institute, Tokyo, Japan; c Hokusetsu General Hospital, Osaka, Japan; d Promed Hospital, Chennai, India; e Wellington Hospital, Wellington, New Zealand; f Liverpool Hospital, Sydney, Australia; g National Heart Centre, Singapore; h Shanghai Zhongshan Hospital Shanghai, China; i Guangdong General Hospital, Guangdong, China; j Guangdong Provincial People’s Hospital Nanhai Hospital, Guangdong, China; k Beijing Fuwai Hospital, Beijing, China; l Asan Medical Centre, Seoul, South Korea; m National Taiwan University Hospital, Taipei, Taiwan; n Toyohashi Heart Centre, Toyohashi, Aichi, Japan

**Keywords:** 3-dimensional wiring, chronic total occlusion, percutaneous coronary intervention

## Abstract

Antegrade wiring (AW) is the prevailing chronic total occlusion (CTO) crossing technique. For proximal cap ambiguity, the Global CTO consensus group uses the “anatomy dictates strategy” method: 1) intravascular ultrasound; 2) move the cap; or 3) retrograde. For CTO body crossing, anatomy dictates 4 strategies: 1) CTOs with tapered stump—loose tissue tracking; 2) CTOs with clear intimal path—intentional intimal tracking with 3-dimensional (3D) wiring; 3) CTOs without a clear intimal path—intentional intimal tracking with intermediate penetration wire; and 4) the “long plus CTOs”—intentional subintimal wiring. The new angiographic 3D antegrade puncture technique from the APCTO (Asia Pacific Chronic Total Occlusion) Club is presented for distal cap puncture. angiographic 3D antegrade puncture technique can be used as a 3D wiring technique as well as an antegrade dissection and re-entry technique. Based on these new frontiers, we have updated our APCTO algorithm in this paper. This update can form a basis for research and training.

## Highlights

• AW is the main method of CTO crossing, with many advances made recently.
• New methods of 3D and adapt wiring with the new APCTO algorithm can improve success.
• The review brings out new techniques in each step of AW.
• Future direction of research for CTO PCI and training can be based on this paper.

Antegrade wiring (AW) is the prevailing technique to cross chronic total occlusions (CTOs). For nondedicated CTO operators unfamiliar with Stingray (Boston Scientific) and retrograde CTO percutaneous coronary intervention (PCI), AW is the only method for CTO crossing. For dedicated CTO operators, over the past decade there has been an increasing use of AW because of improvement of operator’s wire skills and wire technology, leading to a decline in Stingray and in retrograde use in the CTO registries.^1^, ^2^, ^3^, ^4^, ^5^, ^6^, ^7^ Therefore, AW remains the most used CTO technique around the world. There have been many new techniques and technologies for AW in recent years, especially in the Asia Pacific area relating to 3-dimensional (3D) fluoroscopic and intravascular ultrasound (IVUS) wiring. The APCTO (Asia Pacific Chronic Total Occlusion) Club, a group of 14 high-volume CTO operators recognized as leaders in CTO intervention in their respective countries, present the new frontiers in AW. This paper serves as an important update to the previous APCTO Club algorithm and wiring methodology (Central Illustration), and we review the recent advances in AW and present the new techniques and approaches of the APCTO Club, in particular the angiographic 3-dimensional antegrade puncture technique (ADAPT). The paper will follow the CTO wiring by discussing first proximal cap, then CTO body crossing, and finally distal cap puncture.

**Central Illustration:**
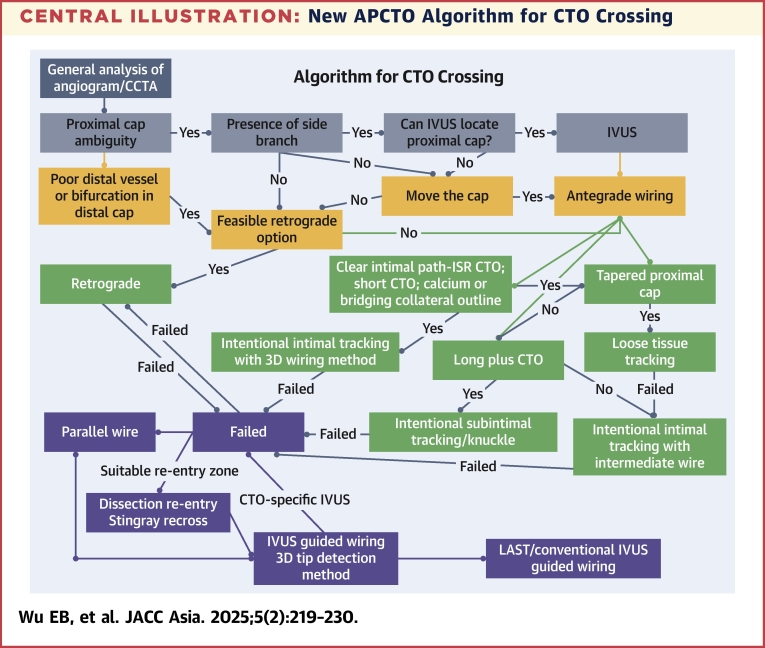
New APCTO Algorithm for CTO Crossing Analysis of angiogram and coronary computed tomography angiography (CCTA) identifies proximal cap ambiguity; if there is a side branch, intravascular ultrasound (IVUS) examination is recommended. If IVUS cannot locate the proximal cap and there is feasible retrograde option, we should go retrograde. If not, we go to move the cap. If IVUS fails to puncture the cap, go to move the cap. Poor distal target or bifurcation should be resolved by retrograde. Antegrade wiring: in clear intimal path, go to intentional intimal tracking with 3-dimensions (3D), and if it fails, we go to dissection re-entry or parallel wiring and finally to IVUS-guided 3D tip detection and limited antegrade subintimal tracking (LAST). If no clear intimal path but a tapered cap, go to loose tissue tracking and if that fails to go toward intentional intimal tracking with intermediate penetration wire. In long plus chronic total occlusion (CTO), we should go to intentional subintimal tracking and go toward dissection re-entry. ISR = in-stent restenosis.

## Puncturing the Proximal Cap

Most proximal caps have a clear tapered stump and can be wired by intermediate penetration force wires. However, 20% to 35%^6^^,^^7^ of proximal caps are difficult because of either ambiguity or tough proximal cap.

## The Ambiguous Proximal cap

There has been a clear advance and global agreement on how to deal with proximal cap ambiguity (PCA) over the past 12 years. The first CTO algorithm, the hybrid algorithm,^8^ suggested the use of retrograde CTO PCI to remove PCA. The APCTO Club introduced the use of IVUS to remove PCA.^9^ The EuroCTO club further improved upon this in 2019^10^ by advising the use of a step wise methodology: 1) IVUS-guided proximal cap visualization; 2) balloon-assisted subintimal entry (BASE) and scratch and go techniques; and 3) retrograde to remove PCA. Finally, in 2021, the Global CTO PCI consensus group, consisting of 125 authors from 50 countries, provided an agreed methodology^11^ that uses the important principle of “anatomy dictates strategy” to determine the best method of overcoming PCA in each CTO. In total, 11% of PCA cannot be overcome with IVUS (JCTO registry, unpublished data), and 50% do not have feasible retrograde option and cannot be resolved with retrograde techniques. Therefore, the anatomically appropriate technique should be used to overcome PCA. Computed tomography coronary angiography (CTCA) is increasingly used to resolve PCA and has been shown to be useful in 27% of cases.^12^ For every proximal cap there is “the best” method of removing ambiguity. If retrograde in needed for other reasons, such a bifurcation in the distal cap then retrograde removal of ambiguity is the obvious choice. If a CTCA is available before the procedure, then careful analysis of the CTCA can be the most useful method to resolve ambiguity.

## The Tough Proximal Cap

### Wire puncture

**Figure 1:**
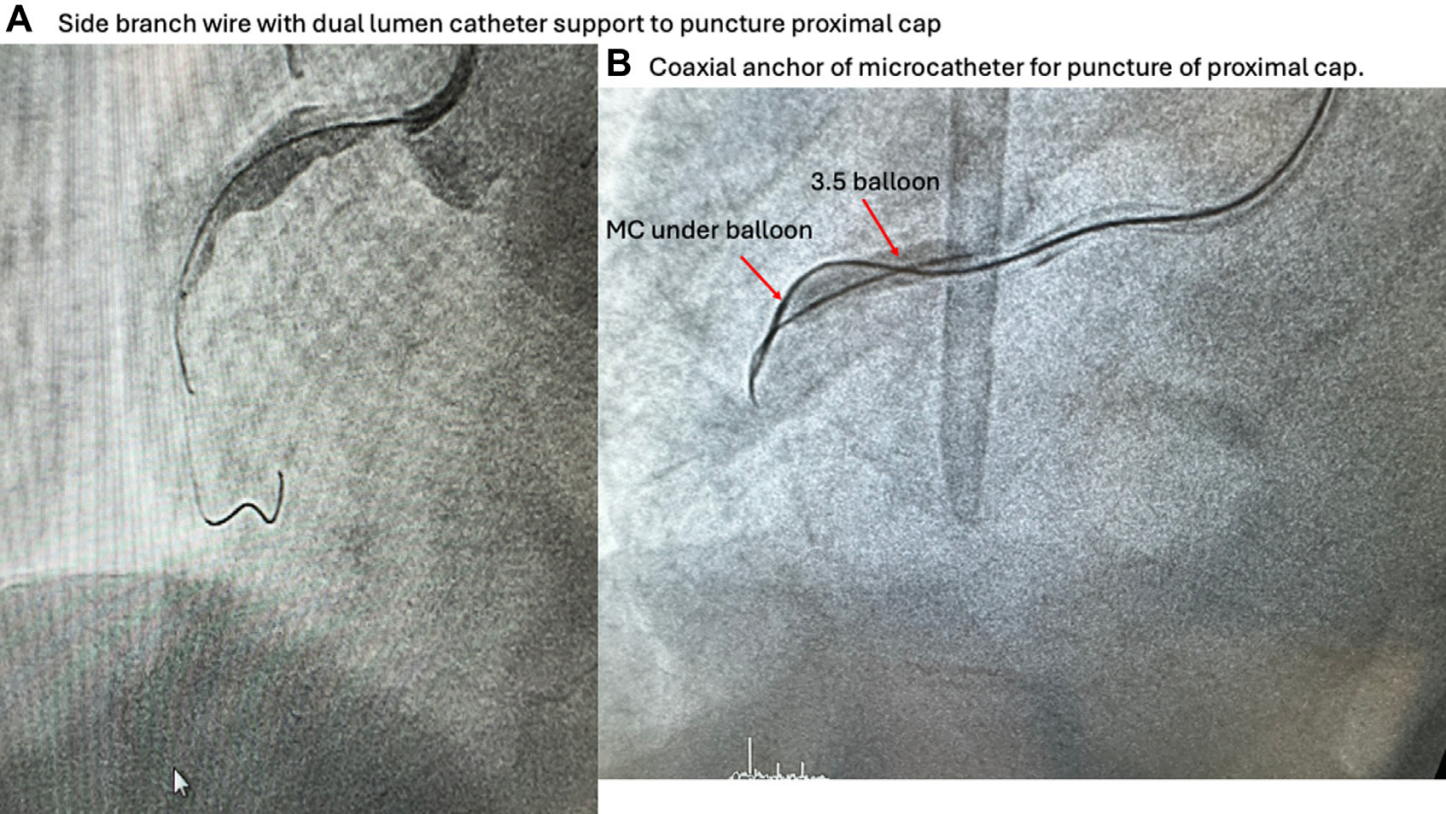
Increasing Backup Support (A) Side branch dual lumen catheter support to puncture proximal cap. Wire in side branch with dual lumen catheter loaded onto the side branch wire to support a chronic total occlusion wire to penetrate the mid right coronary chronic total occlusion. (B) Coaxial anchor of microcatheter (MC) for puncture of proximal cap. MC under a 3.5-mm balloon that is trapping the MC in a coaxial fashion to increase support for a chronic total occlusion wire placed inside the MC to penetrate the chronic total occlusion proximal cap.

After resolving ambiguity, there remains a proportion of proximal cap that are tough and the use of high-penetration force wires, such as Conquest/Confianza 12 g or 8/20 (Asahi Intecc), Hornet 14 (Boston Scientific), or HI-Torque Progress 200T (Abbott Vascular), and special backup force methods are needed. Once again, anatomy dictates strategy, and use of a dual-lumen catheter where a side branch is present (Figure 1A) or coaxial balloon anchoring of antegrade microcatheter where proximal vessel allows for it (Figure 1B). Extraplaque techniques such as BASE or proximal cap modification by subintimal ballooning can be useful to bypass the tough proximal cap.

### Delivery of devices

Recent publications have provided stepwise guidance on how to overcome an impenetrable proximal cap through intraplaque and extraplaque techniques once wire entry is achieved.^13^ Intraplaque techniques essentially use increasing backup force and penetration force of the wire to achieve intraplaque penetration through calcium. It is strongly recommended to increase backup force first before increasing penetration force of the wire. Back up force can be increased with using supportive guiding catheters such as Amplatz guide, use of side branch anchoring technique, use of a buddy wire in the side branch anchor to increase friction, coaxial balloon anchor (Figure 1B), guide extension catheter, and more recently, the grip technique, where the guide extension itself is anchored with a coaxial balloon to provide extreme support. Penetration force of the wire can be increased with stepping up to Confianza Pro 12 (Asahi Intecc), Hornet 14 (Boston Scientific), Infiltrac Plus (Abbott Vascular), Warrior (Teleflex), Astato 20/40 (Asahi Intecc), Conquest Pro Sharpened tip CPST (Asahi Intecc), or Conquest 8/20 or 8/40 (Asahi Intecc).

Extraplaque techniques include all of the "move-the-cap" techniques (eg, BASE, scratch-and-go, side BASE, Carlino technique, side branch lithotripsy) and require antegrade dissection and re-entry (ADR) approaches to re-enter, which are described later in this paper.

## Crossing the CTO Body

There are 3 described methods for CTO body crossing: loose tissue tracking, intentional intimal tracking, and intentional subintimal tracking.^14^ The new frontier in CTO body crossing is to use the “anatomy dictates strategy” method and decide up front which method of wiring is to be used. We do not need to stick rigidly to this decision, but some sort of decision should be made regarding which method to use based on the anatomy. We recognize 4 approaches based on anatomy: 1) CTOs with tapered stumps or residual channels should be approached with loose tissue tracking and soft tapered tip wires such as XTA (Asahi Intecc), Bandit (Teleflex, Wayne PA USA), or Fighter (Boston Scientific); 2) CTOs with clear intimal path (ie, short CTOs, in-stent restenosis [ISR] CTOs, and heavily calcified or clear antegrade bridging collaterals that outline the vessel, or CTO with prior patent vessel angiogram) should be approached with intentional intimal tracking and wired with 3D wiring (if fluoroscopic angles allow) using Gaia Next 3 or 4, conquest 12 (Asahi Intecc); 3) CTOs without taper stumps and without a clear intimal path should be wired with intentional intimal tracking without 3D wiring using intermediate penetration force blunt tip wires such as UB3, Neo 3, Gladius EX (Asahi Intecc), or Pilot 200 (Abbott Vascular); the Gaia Next 2 (Asahi Intecc) or above wires should not be used if there is ambiguity of vessel course. The intention here is to track intimal path while acknowledging the chance that the wire may move subintimally; and 4) the “long plus CTOs” with ambiguous course, tortuosity, and heavy calcium should be approached with intentional subintimal wiring (Central Illustration). On balance, if we have a calcified CTO where the intimal plaque path can be seen, knuckle wiring should be preferred in those longer, more tortuous, and more heavily calcified CTOs, whereas shorter, less tortuous ones can be approached with 3D wiring.

## Loose Tissue Tracking

Previously, the “loose tissue tracking wires” such as XTA wire, fighter, or Bandit have been used for loose tissue tracking, and they remain a good option for many cases. However, the Gladius EX wire (Asahi Intecc), a polymer jacketed 3-g tip load nontapered wire, can also perform loose tissue tracking when a little more penetration force is needed and is often a better first-choice wire for loose tissue tracking.

“Intentional” intimal tracking and “intentional” subintimal tracking are strictly intentional, because we all know that sometimes a knuckle wire travels in the intima and goes true to true and other times “intentional” intimal tracking turns out to be subintimal, and the only way to know is to perform IVUS imaging after CTO crossing.

## Intentional Subintimal Tracking

Intentional subintimal tracking has been performed with knuckle wiring, and the recent advance of the Gladius MG (Asahi Intecc), a preshaped 3-g wire with modified distal core that allows prolapse suitable for knuckle wiring, allows for more small knuckle wiring. Therefore, we currently have the option of having a big knuckle if we use a Pilot 200 or a small knuckle if we use the Gladius MG. The bigger knuckle makes for a bigger subintimal space but is less likely to run into small side branches, and so we need to be wary of the risk of the smaller knuckle of the Gladius MG running into a small side branch and be diligent in checking multiple views to ensure that the knuckle travels in the main vessel when using it. The use of intermediate penetration force blunt-tip wires, such as Pilot 200 (Abbott Vascular), Neo 3, UB 3, and Gladius EX (Asahi Intecc), allows for intentional subintimal tracking without the use of knuckle. Knuckle wiring should be done up front in cases where there is long ambiguous segment, heavy calcium, and tortuosity, the so called “long plus” CTO. Hematoma formation is a significant risk for intentional subintimal tracking, which can compress distal lumen complicating ADR. Careful management of the subintimal space is needed. Inflow protection with the use of a guide extension catheter is an excellent way to control subintimal space expansion. Routine decompression of the subintimal space by aspiration on the Stingray balloon during preparation of wire exchange and use of bob-sledding can reduce subintimal space expansion. The Subintimal TRAnscatheter Withdrawal (STRAW) technique^15^ can be used if there is already subintimal space expansion, and this can be upgraded by using a 1:1-sized balloon to occlude the proximal vessel, allowing for a complete STRAW. STRAW can also be performed during stenting by connecting an empty indeflator to a low-profile microcatheter (such as fine cross [Terumo]) placed in the subintimal space during stenting and stent optimization to pull back blood from the extraplaque space—the extraplaque blood withdrawal technique.^16^

## Intentional Intimal Tracking

Intentional intimal tracking requires accurate knowledge of the intimal position and is possible when there is a clear fluoroscopic path as to the intimal position, such as in ISR CTO or heavily calcified CTO where the calcium outlines the intimal plaque position or prior angiogram before coronary occlusion. Placement of an IVUS catheter into the subintimal space can also allow for a clear location of the intimal position and allow intentional intimal tracking. In theory, if CTCA can be of high enough resolution and be correlated to the angiographic views with an accurate software, CTCA-correlated intentional intimal tracking can be done in the future. However, currently intentional intimal tracking is only useful in ISR, previous angiogram of patent vessel, short clear-course CTOs, and heavily calcified vessels.

In the recent few years, because of the development of wires that can deliver an accurate degree of torque to the tip such as Gaia next series (Asahi Intecc), there has been a move toward 3D wiring. It is obvious that the wire within a coronary artery is a 3D object within a 3D structure, but we are processing the information from a 2-dimensional fluoroscopic output. However, by using appropriate angles, we can reconstruct a 3D image allowing for 3D wiring. Okamura et al^17^ first described a 3D method and subsequently demonstrated the technique to be faster, safer, and more successful in retrospective registry data.^18^ At the same time, the group developed the “same in front opposite behind” principle.^18^ Others have managed to reproduce success with this method even in cases where the wire has gone subintimal,^19^ but the adoption of 3D wiring globally has been very limited because of the difficulties in understanding the principles and formation of a mental 3D image. Wu, Nagamatsu, and Okamura^20^ have recently distilled the essence of 3D wiring into 10 tips that can allow an operator to gain most of the benefits of 3D wiring without actually forming a mental image or doing 3D wiring. The APCTO Club has also developed a method for 3D wiring without the need for mental image called ADAPT wiring, and these techniques can also be used for intentional intimal tracking. We will return to this method after the full description of ADAPT wiring. This break in this frontier of AW means that the APCTO Club will change their original recommendation of using Crossboss (Boston Scientific) for crossing ISR CTO^9^ to using 3D wiring for ISR CTO.

## Conclusions in CTO Body Crossing

The aim of wiring through a CTO body: 1) get to the distal cap; 2) avoid wire perforation outside the vessel architecture (these 2 are essential); 3) avoid wire going into the subintimal space; 4) avoid enlarging the subintimal space and forming hematoma; and 5) avoid enlarging the periwire space, which reduces antegrade wire control (these 3 are preferable).^21^ An anatomy dictate strategy method for CTO body crossing should be used separating CTOs into the following: 1) loose tissue tracking (XTA, Bandit, Fighter, Gladius EX); 2) intentional intimal tracking with 3D wiring (Gaia Next 3, Gaia Next 4, Conquest 12); and 3) intentional intimal tracking with intermediate penetration force wires (Gladius EX, Pilot 200, UB3, Neo 3) and intentional subintimal tracking (Gladius MG, Pilot 200, UB3, Neo 3). This is quite a change compared with the recommendation of the APCTO algorithm^9^ with a reduction in the use of Gaia second for CTO body wiring in favor of blunt tip intermediate-force wires. Therefore, we hereby update our algorithm from Gaia second (which was suggested as the CTO body crossing wire after XTA) to Gladius EX/ UB3/Neo 3. For ISR CTO and CTO with visible calcium with clear intimal position, and in future CTCA fluoroscopic-correlated CTOs, intentional intimal tracking with 3D wiring shows great promise.

## Distal Cap Puncture

One of the important frontiers in AW for distal cap puncture is the recognition of the dangers of losing a side branch in CTOs with the distal cap at a bifurcation. The Global CTO consensus group recommended the use of retrograde approach in CTOs where the distal cap is at a bifurcation and there is a feasible retrograde option.^22^ There is also the recognition in the APCTO Club that not all distal caps are the same and some features are highly suggestive of a tough distal cap that requires a step to a high penetration force wire for successful wiring.^21^ These include the rounded distal cap stump, the presence of a large side branch at the distal cap, visible calcium at the distal cap, and sharp tapered distal cap stump. Now, we further propose that if a tough distal cap is seen, we should use 3D wiring techniques for distal cap puncture with a high penetration force wire because it would be safer and faster.^18^

## Angiographic 3-Dimensional Antegrade Puncture Technique

The 3D wiring technique recommended by the APCTO Club is the ADAPT technique.

First, we need to decide which distal cap is worth the investment of time, equipment, and energy in using ADAPT wiring. Once again anatomy dictates strategy. Prerequisites for ADAPT wiring are that the distal cap has to be visible, which can be improved by using selective contrast injections through microcatheters placed in the predominant retrograde channel, and the distal cap should be in a fluoroscopically easy position to be seen from 2 perpendicular 90° angles. The fluoroscopic angles are left anterior oblique (LAO) 45° and right anterior oblique (RAO) 45° in mid right coronary artery distal caps; LAO 30° cranial 45° and LAO 30° caudal 45° in distal right coronary artery distal caps; and LAO 45° cranial 30° and RAO 45° cranial 30° in mid-distal left anterior descending artery distal caps. Full descriptions of other coronary segments are published.^23^ However, even when the prerequisites are reached, we should still consider the anatomy. The presence of features of a tough distal cap, the presence of a bifurcation at the distal cap, failure for first AW attempt, and the lack of a feasible retrograde option are all indications for ADAPT wiring.

## The Set Up for ADAPT Wiring

For ADAPT and indeed any type of 3D fluoroscopic wiring, we need the following:

1. Stop wiring when the wire is 10 mm from the target, in this case the distal cap. It is easier to wire through virgin territory where the wire space is tight and not enlarged, and so we should stop 10 mm before the distal cap.
2. Drill the microcatheter into the CTO body to increase backup support. If the microcatheter cannot be passed through the proximal cap we should “break the cap” with a small balloon and re-exchange back to the microcatheter.
3. Switch out for a high-penetration force wire (Gaia Next 4 wire, conquest 12 g, and in development, a 15-g preshaped dedicated distal cap penetration wire) with a 45° bend at 1 mm from the tip and use a torque device on the wire, because precise rotational angles are required for ADAPT. A special “Sake Cup Torque augmentation” device is being developed for this purpose (Figure 2).

**Figure 2:**
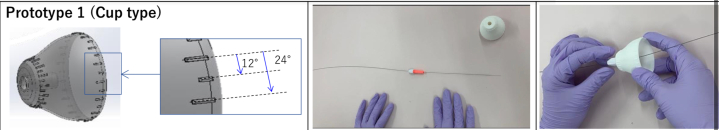
“Sake Cup Torque Augmentation” Device A prototype 5-cm torque device with 12° markings to allow accurate torque of 12°.
4. Set up the corresponding 90° fluoroscopic views and perform cine angiography with retrograde contrast injection.

## Wire Manipulation for ADAPT

**Figure 3:**
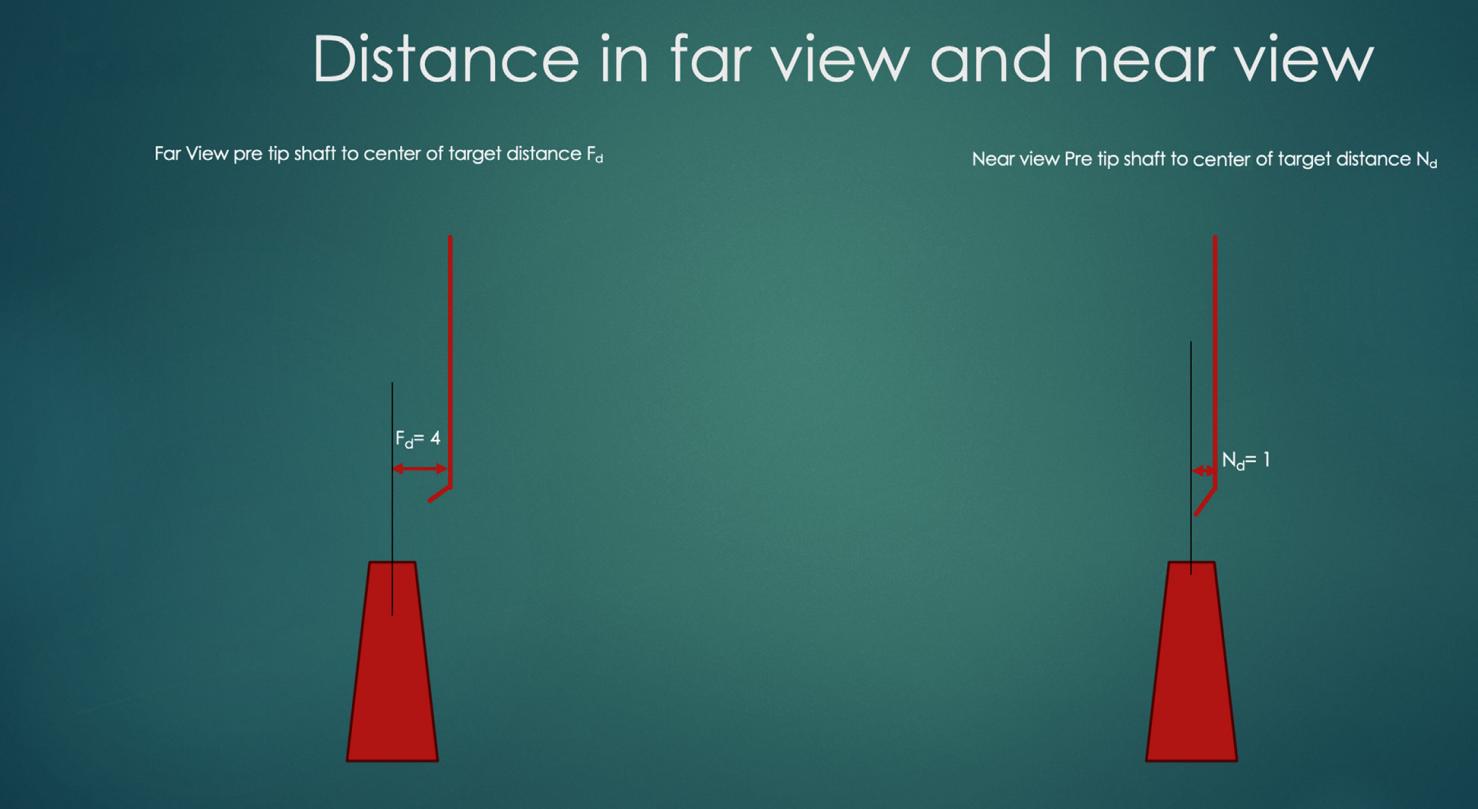
Distance in Far and Near View Far view distance (Fd) is measured from the pre tip shaft to the line drawn from the midpoint of the distal target up the vessel. On the right, is the near view and the near view distance (Nd) defined as the distance between the pre tip shaft and the imaginary line drawn from the middle of the distal target up the vessel.

The steps for ADAPT are as follows:

1. Identify the view where the shaft of the wire proximal to the tip is further from the central point of the distal cap target, and in that view (called the far view), rotate the wire tip to point toward the target.
2. In the near view, try to straighten the wire tip by miniscule rotation. This manipulation will let us know which rotation direction (clockwise or counterclockwise) will lead to the tip moving toward the target. Straightening the wire tip will also make the next rotation more precise.
3. We need to measure the horizontal distance from the pretip shaft of the wire to the imaginary projected line from the center of the distal cap to the same longitudinal distance of the wire tip in both the near view and the far view (Figure 3). We then calculate the ratio of the distance in the near view divided by the far view. From this ratio we can deduce the angle of rotation needed to perfectly align the wire to the target. If the ratio is 1:4 we should rotate 11°, if it is 2:4 we should rotate 22°, if 3:4 we should rotate 33°, and if the distance is the same (ie, 4:4) we should rotate 44°. For reference, 11° is roughly equivalent to 2 minutes on the clockface, 22° is 4 minutes, and 44° is 7.5 minutes on the clock face. We can see that these small rotations require a torque device for precise manipulation of the wire.

**Figure 4:**
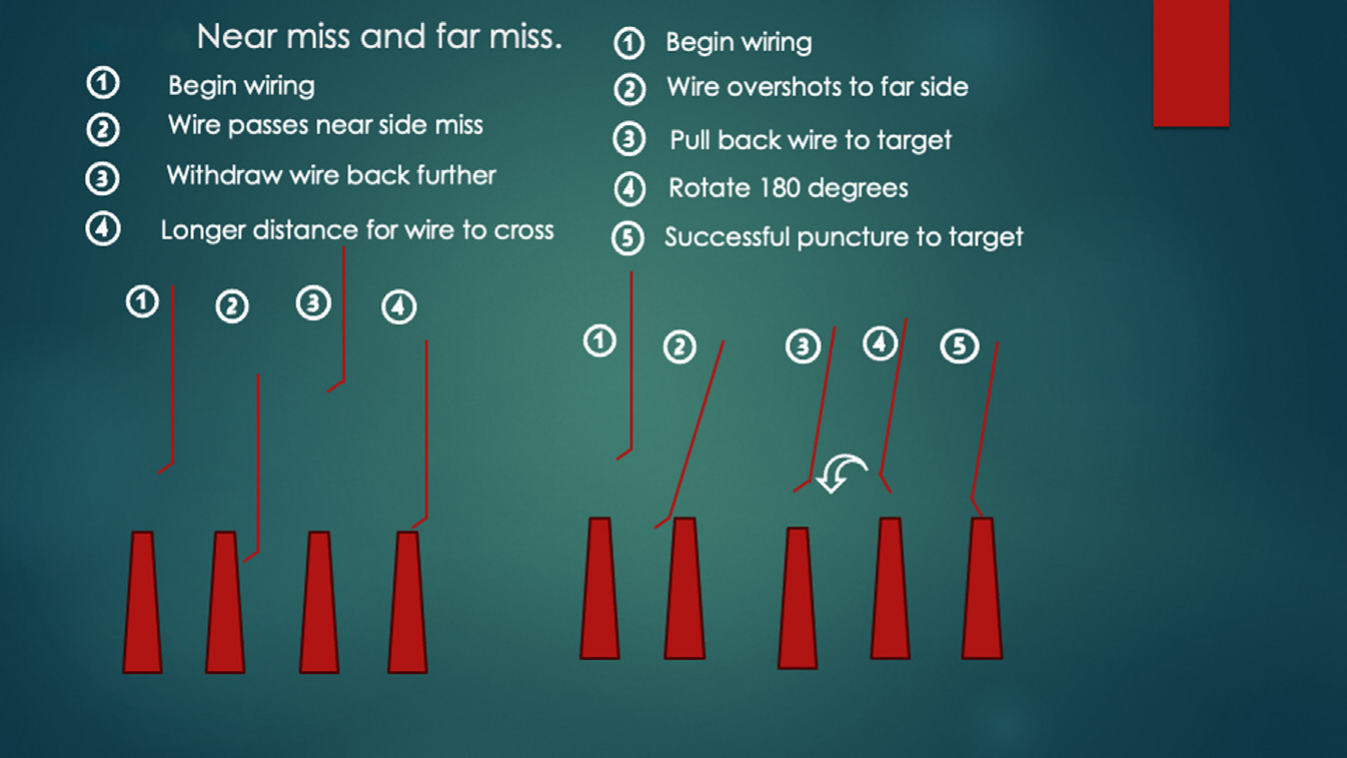
Near and Far Miss On the left: 1) begin wiring; 2) the wire passes beyond the chronic total occlusion target on the near side (near to the wire); 3) the solution for near miss is to withdraw the wire further proximally to allow distance for the wire to transverse horizontally; and 4) the wire then can get to target. On the right: 1) begin wiring; 2) wire overshoots the chronic total occlusion target toward the far side (far to the wire side); 3) the solution for far side miss is to pull back the wire toward the target; 4) rotate 180° and then push forward to; and 5) repuncture the target.
4. Distal cap puncture. After rotation, we should go to the far view to observe the forward puncture of the wire into the distal cap.

## Near and Far Miss in ADAPT

Even if the wire tip is rotated precisely to point at the distal cap, the wire tip can still miss the target through 2 mechanisms: near miss and far miss. Near miss is when the wire travels too far longitudinally without crossing horizontally, leading to the wire missing the target on the near side. The solution to near miss is to allow the wire more longitudinal distance so that it has the length to travel across to the target. To do this, we must pull the wire back proximally without rotation to more proximal position and puncture forward again (Figure 3). The other miss is the far miss. In far miss, the wire travels horizontally too much and crosses the target before it is at the horizontal site of the distal cap (Figure 4). The solution in this case is to pull back the wire until the tip is just above the distal cap and rotate 180° so that the wire tip points down at the distal cap target and push forward.

Once the wire punctures into the distal cap, true lumen position can be confirmed with retrograde injection, and after gaining a small distance, the microcatheter can be passed to the distal true lumen and the high penetration force wire can be switched out to a workhorse wire.

ADAPT provides a way to capture all the information from 2-dimensional fluoroscopic views to effectively perform 3D wiring without the need for a mental image and with very precise rotation angles. We recommend the use of this technique in tough distal caps, bifurcation distal caps without retrograde options, and failed first AW attempts. Due to the use of high-penetration force wire and precise direction of the wire tip, ADAPT can penetrate back into true lumen even from subintimal space and is a type of ADR.

Returning briefly to CTO body crossing under intentional intimal tracking, one can see that ADAPT can be used as a 3D wiring method for intentional intimal tracking in ISR CTO or calcific CTO. This new method we believe provides a 5-step simple and learnable method for easy 3D wiring: 1) set up identical to the first 4 steps of ADAPT wiring, with wiring only 5 to 10 mm with each step, using high-penetration force wires, keeping the microcatheter support strong, and using 90° apart specific fluoroscopic angles for particular coronary segments; 2) identify the far view and rotate the wire tip toward the target—which is defined as the center of the vessel; 3) go to the near view and straighten the wire tip, which will let us know which direction will rotate the wire tip toward the center of the vessel; 4) in the near view, rotate the wire tip until it just begins to move toward the target and then push forward observing the wire in the far view; and 5) stop when the wire tip gets to the center of the vessel in the far view or has travelled 5 mm and reassess again. Precise angles of rotation as per ADAPT wiring are not necessary because we are not aiming for a precise target, but just to be more central in the vessel. This method does not require any mental map nor any mental calculation at all and can be used to reproduce very high-quality 3D wiring.

## The Mathematical Trigonometry Model Behind ADAPT Wiring

**Figure 5:**
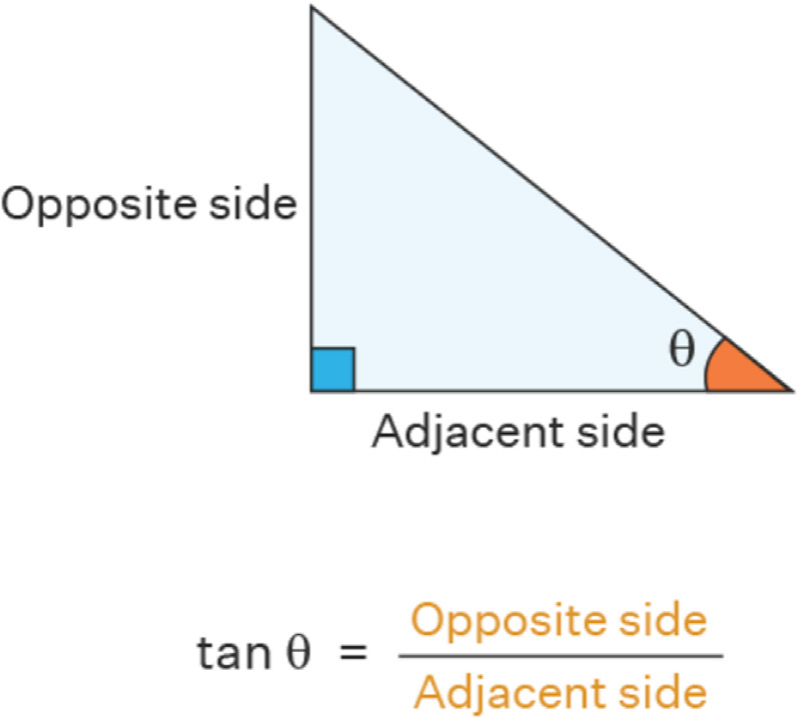
A Right-Angle Triangle With Tangent Θ = Opposite/Adjacent The classic right angle triangle with the opposite side and adjacent sides labelled. The angle θ is calculated by the formula: tangent θ = opposite/adjacent.

**Figure 6:**
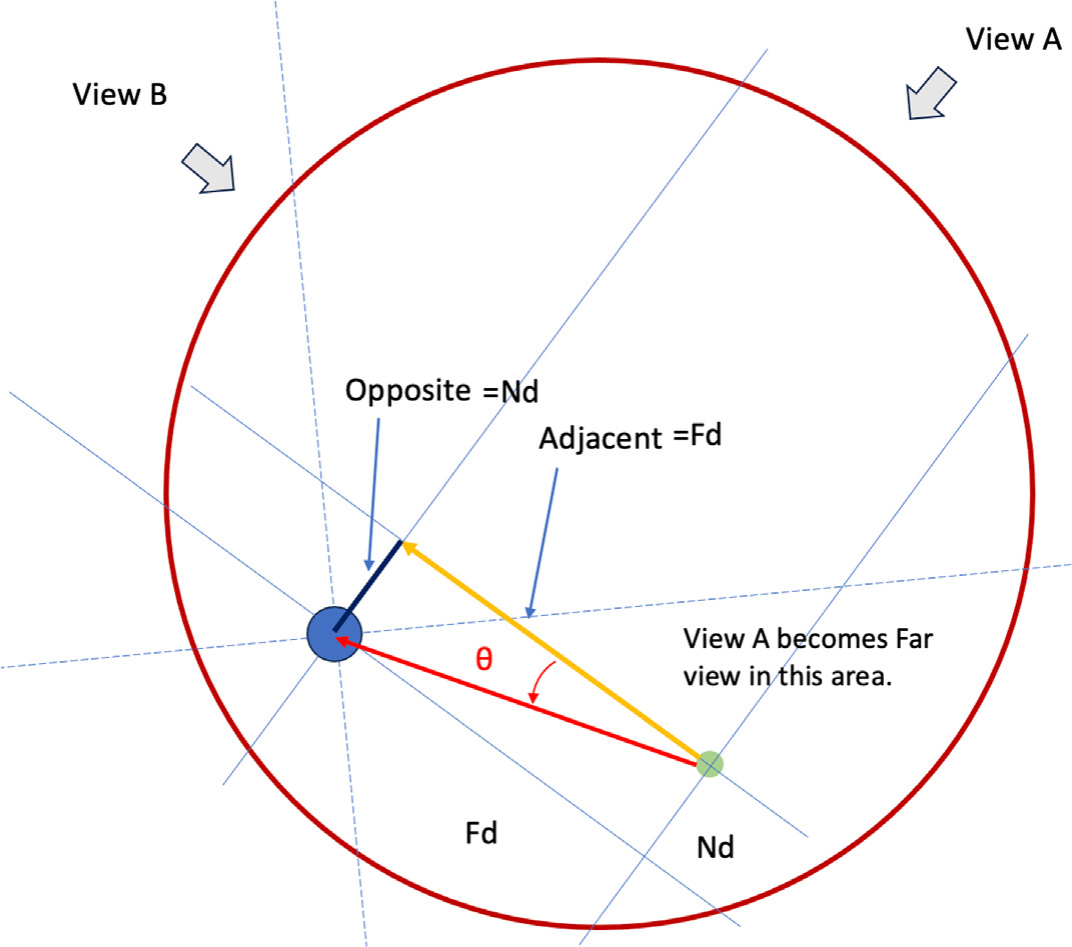
Near View Distance/Far View Distance = Tan Θ In this diagram of a coronary artery (red circle), which is observed via 2 angiographic views at 90° apart (view B and view A), the wire position (green dot) is far from the chronic total occlusion distal true lumen target (blue dot). In view B the distance of the wire to the distal target (black line) is less and thus view B is the near view, and the distance is the Nd, which corresponds to the opposite in the right angle triangle. Similarly, view A is the far view and the distance between the wire and the distal target in view A is the Fd (yellow line), which corresponds to the adjacent of the right angle triangle. Therefore, the angle θ, the angle the wire has to rotate to face the target straight on, is calculated simply as tangent θ = Nd/Fd. Abbreviations as in Figure 3.

**Figure 7:**
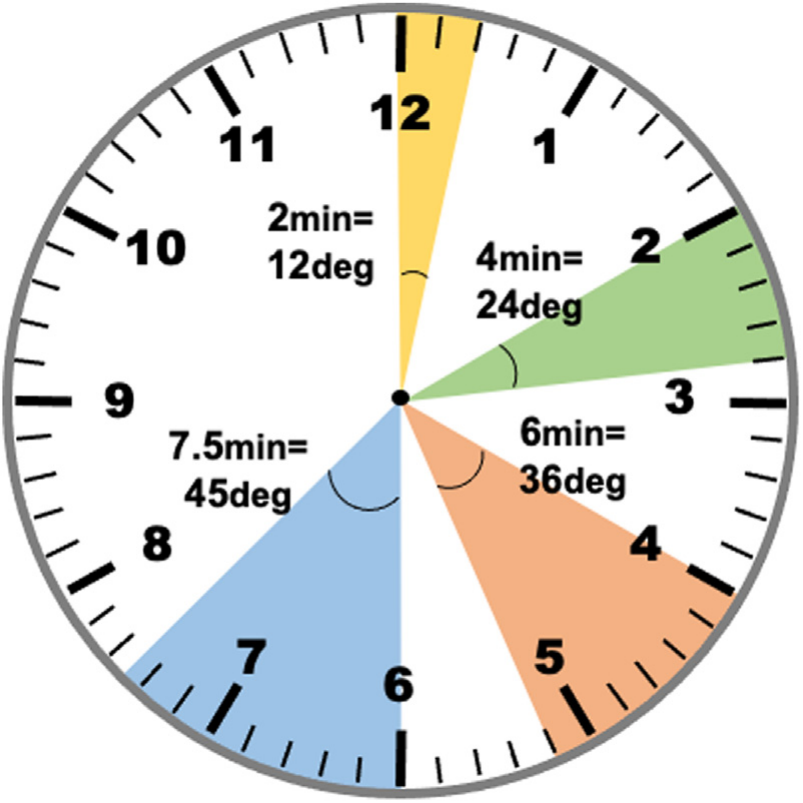
Clockface Minutes Relating to Angle of Rotation This clock face illustrates the angles corresponding to minutes on the clock face. From 12 o’clock to 2 minutes is 12° (yellow); from 2 o’clock to 14 minutes past the hour is 4 minutes and corresponds to 24° (green); from 4 o’clock to 26 minutes past the hour is 6 minutes and corresponds to 36° (pink), and from 6 o’clock to 37.5 minutes past the hour is 7.5 minutes, which is 45° (blue).

ADAPT wiring is based on the simple trigonometry model that in a right-angle triangle, the angle θ can be derived from the formulae tangent θ = opposite/adjacent (Figure 5). By measuring the distance between the wire shaft and the target in the near view (Nd), where the wire is closer to the target, and dividing this by the distance between the wire shaft and the target in the far view (Fd) with the fluoroscopic views 90° apart, we can form a right-angle triangle and the Nd will be the opposite and the Fd will be the adjacent. By performing the calculation tan^−1^ of Nd/Fd, we can obtain the angle θ (Figure 6). One can calculate this on a scientific calculator with the function tan^−1^ (Nd/Fd) = θ, giving tan^−1^ (1/4) = 14; tan^−1^ (2/4) = 26.6; tan^−1^ (3/4) = 36.9; and tan^−1^ (4/4) = 45. Therefore, the wire should be rotated by 14° if the Nd/Fd is 1/4; 27° if Nd/Fd is 2/4, 37° if Nd/Fd is 3/4, and 45° if Nd = Fd. Our proposal of 11° (for Nd/Fd 1/4), 22° (for Nd/Fd 2/4), 33° (for Nd/Fd 3/4), and 44° (for Nd/Fd 4/4) is used because it is much easier to remember, and smaller degrees for each Nd/Fd might prevent the tendency to over rotate the wire. If we use the clock face method to understand the amount we have to rotate, 2 minutes on the clockface is 12°, 4 minutes is 24°, 6 minutes is 36°, and 45° is 7.5 minutes on the clock face (Figure 7). Table 1 illustrates the differences between the 11,22,33,44 method and the clockface method comparing to the actual θ and the difference in angle comparing the methods are expressed in parenthesis.

**Table 1:** Differences Between Actual θ and Clockface vs 11,22,33,44 Angles

Nd/Fd	θ	11,22,33,44 Rule	Clock, min	Clock Degree
1/4	14	11 (−3)	2	12 (−2)
2/4	27	22 (−5)	4	24 (−3)
3/4	37	33 (−4)	6	36 (−1)
4/4	45	44 (−1)	7.5	45 (0)

**Figure 8:**
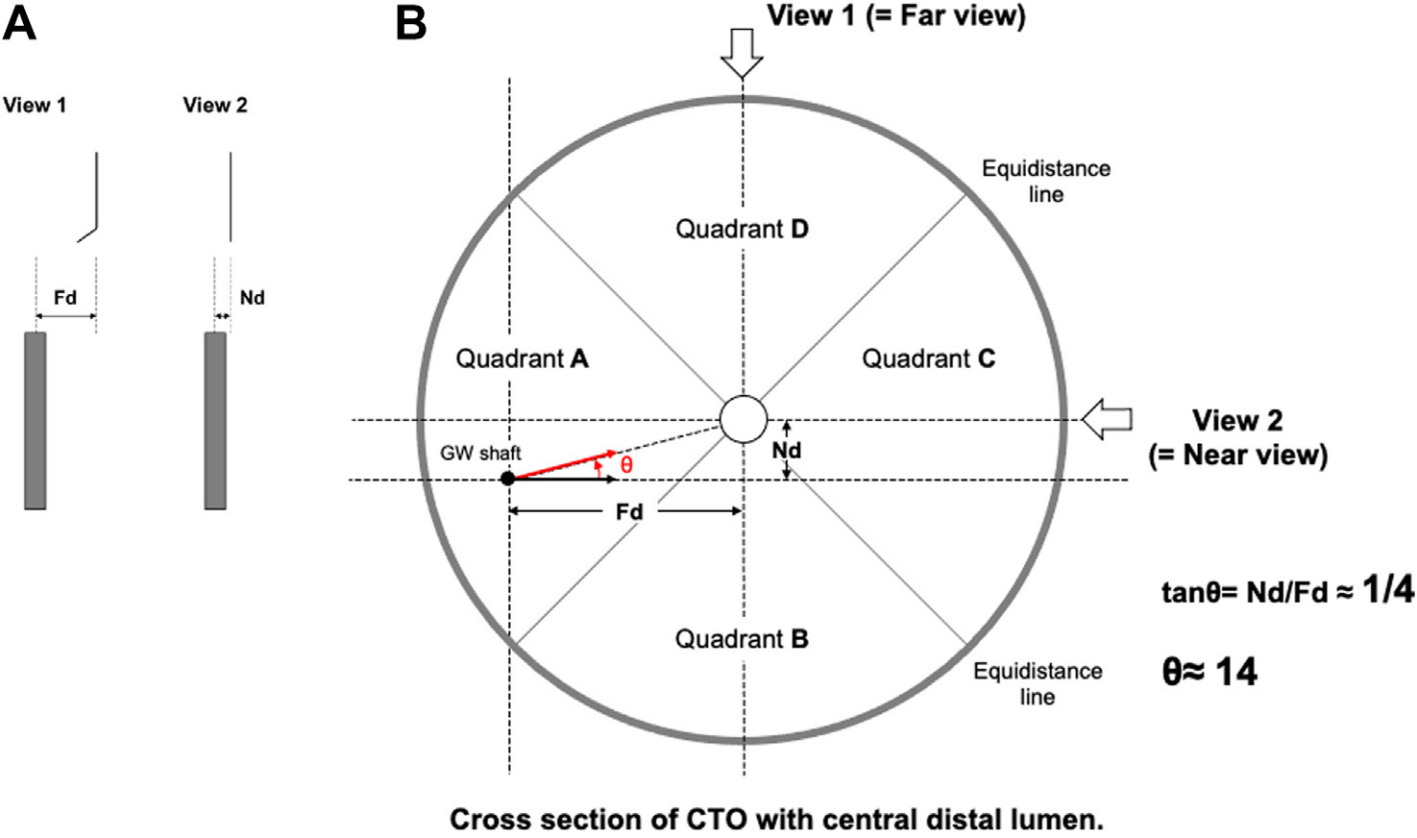
Understanding Far and Near View: Cross Section of CTO With Central Distal Lumen (A) We illustrate the measurement of near view distance and far view distance; (B) on the right, a coronary artery (large gray circle) is observed via view 1 far view and view 2 near view. The 2 diagonal lines divides the coronary artery into 4 quadrants. Wires that are on these diagonal lines are equidistant to the central distal target in both near and far views, but wires in quadrants A and C will be further in view 1 than view 2 making view 1 the far view, and conversely, wires in quadrants B and D will be further in view 2 than in view 1. Our wire (small black dot) is located in quadrant A and the Nd is about one-fourth of the Fd and so θ is 14°. GW = guidewire; other abbreviations as in Figure 3.

**Figure 9:**
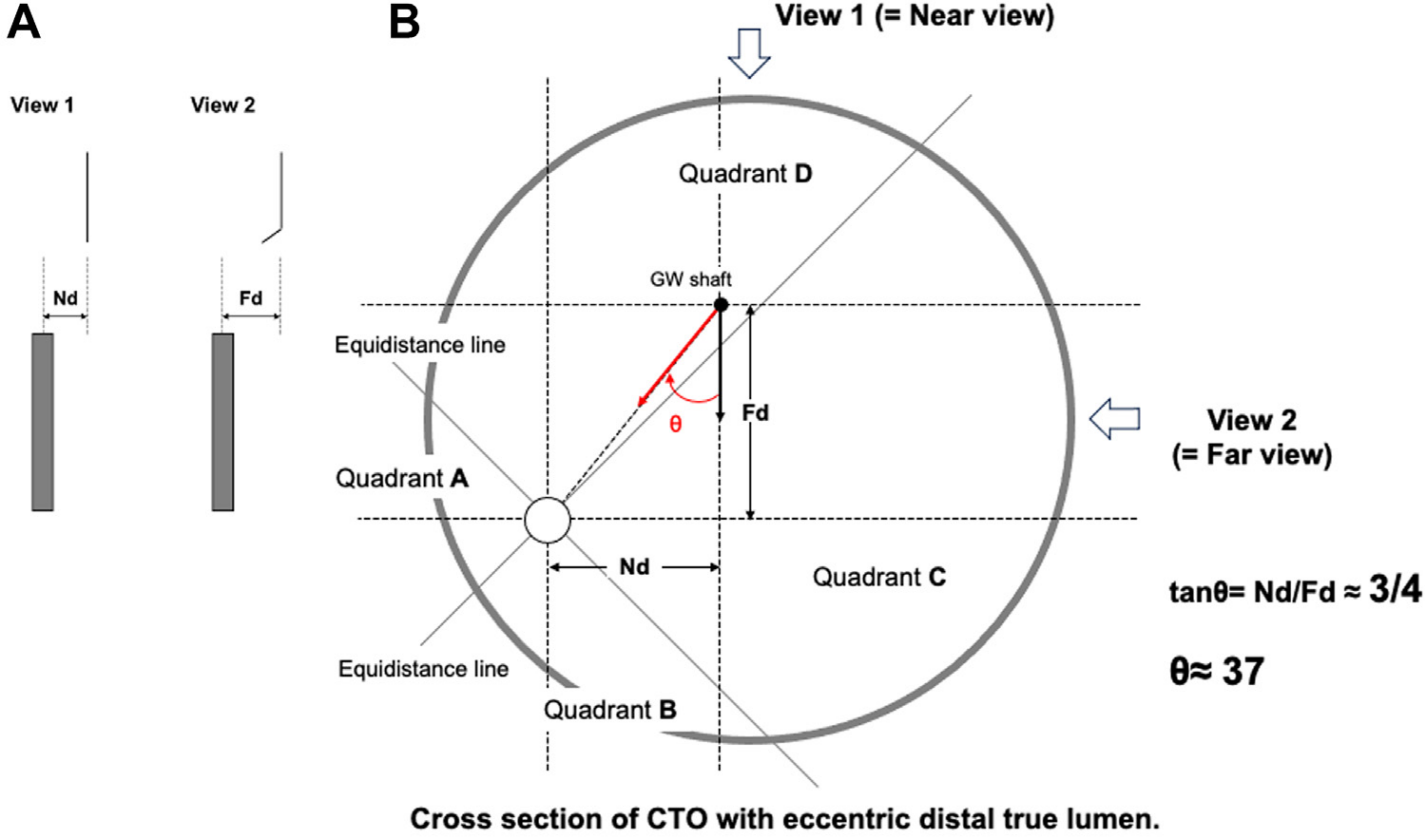
Understanding Far and Near View: Cross Section of CTO With Eccentric Distal True Lumen (A) We illustrate the measurement of Nd and Fd; in (B) on the right, a coronary artery (large gray circle) is observed via view 1 near view and view 2 far view. The distal true lumen is eccentrically located (small circle). The 2 diagonal lines emerging from the distal true lumen divides the coronary artery into 4 quadrants. Wires that are on these diagonal lines are equidistant to the distal target in both near and far views, but wires in quadrants A and C will be further in view 1 than view 2 making view 1 the far view, and conversely, wires in quadrants B and D will be further in view 2 than in view 1. Our wire (small black dot) is located in quadrant D, making view 1 the near view. The near Nd is about three-fourths of the Fd and so θ is 37°. Abbreviations as in Figures 3 and 8.

To understand far and near view, we need to consider the cross-section of a vessel, which is divided into 4 quadrants by 2 straight lines, which are collections of points with equal distances to the target in 2 orthogonal views—the equidistance line (Figure 8). If the wire shaft is in quadrant A or C, the horizontal distance from the wire shaft to the target is shorter in view 2. On the other hand, the horizontal distance from the wire shaft to the target is shorter in view 1 if the wire shaft is in quadrant B or D. In other words, view 1 is the far view and view 2 is the near view in quadrants A and C and view 2 is the far view and view 1 is the near view in quadrants B and D. If we rotate the wire in the far view toward the target and straighten the wire in the near view, with small rotation our wire will point at 3 o’clock in the bold black arrow (Figure 8); therefore, to align the wire tip to the target (bold red arrow in Figure 8), the wire should be rotated by angle θ. Here, tangent θ can be expressed as Nd over Fd (Nd/Fd), in this case 1/4 giving a θ of 14°. If the wire is not central but near the side of the vessel, a similar division into 4 quadrants with 2 equidistant lines can be made (Figure 9) and a similar right-angle triangle can be seen with the Nd/Fd here being 3/4. The θ as calculated by tan^−1^ (3/4) = 36.9, and so a 37° rotation will place the wire tip along the red bold arrow (Figure 9) correctly pointing toward the target. These 2 examples demonstrate the robustness of the ADAPT wiring method.

### Antegrade dissection and re-entry

When the wire performing AW is in the subintimal space, some sort of ADR is needed to enter the distal true lumen. Stingray and now the ReCross (Biotronik) can both do ADR, with Stingray providing a more robust and reliable re-entry. ReCross is a dual lumen catheter with dual, completely separate, over-the-wire lumens and 3 distal exit ports, allowing it to perform parallel wiring, ADR, unique complex PCI procedural techniques as Antegrade Dual Access, and simultaneous subintimal hematoma withdrawal during antegrade wiring. Two other techniques are worth mentioning as “new frontiers.” Carlino’s Subintimal Antegrade Fenestration and Re-entry method seems to work better in cases with less calcium and soft plaque.^24^ Another new frontier is the development of the Simultaneous 3D live IVUS tip detection ADR method using the Anteowl IVUS (Terumo).^25^ The use of the new Conquest Pro 12 Sharpened tip (CP12ST) wire (Asahi Intecc) with a tip sharpened to 0.005 inches, giving it twice the penetration force of conquest 8/20 wire, allows for IVUS guided re-entry from subintimal space into the true lumen to complete IVUS tip detection ADR.^26^ This provides a much faster and reliable IVUS-guided wiring method compared with the older methods of longitudinal location of exit site and the fluoroscopic and IVUS coregistration method of IVUS-guided wiring. However, the availability of Anteowl IVUS is limited to Japan and Hong Kong thus far, and the alternative IVUS systems are slightly more difficult to use. Our experience shows that live IVUS 3D tip detection is efficient and promising and represents a new frontier in AW. However, when in AW we reach the point of considering ADR, we should also at the same time consider whether a switch to the retrograde approach would be faster, safer, and more successful.

## Conclusions

AW remains the prevailing method for CTO crossing. We divided AW into proximal cap entry, CTO body crossing, and distal cap puncture, looking at the advances made in the past 5 years as well as new frontiers that are developing in AW. In proximal cap entry, we described the coming together of global consensus on how to deal with ambiguous proximal cap using the anatomy dictates strategy concept. In CTO body crossing, we highlighted the increasing importance of intentional intimal tracking and 3D fluoroscopic wiring. In distal cap puncture, we described the new APCTO ADAPT method and various new ADR methods.

This paper forms an important update for AW (and the APCTO algorithm) and forms a basis for continued discussion in research, training, and CTO PCI.

## Financial Support and Author Disclosures

Dr Wu has received honorarium for consulting for Boston Scientific and Abbott Vascular; has served on the Board of Directors for APCTO Club Ltd, Hong Kong, which receives research funding from Abiomed, OrbusNeich, and Asahi Intecc; and has received educational grants for conferences from Biotronik, Abbott Vascular, OrbusNeich, Medtronic, and Biosensor. Dr Matsuno has received remuneration for lectures from Asahi Intecc, Boston Scientific Japan, Orbus Neich Medical, and Abbott Medical Japan. Dr Nagamatsu has received consultant honoraria from Asahi Intecc and Abbott Vascular Japan. Dr Kalyanasundaram has received fees from Abbott Vascular, Boston Scientific, Asahi Intecc, and Terumo. Dr Harding has received speaking and proctoring honoraria from Boston Scientific, Abbott Vascular, Bio-Excel, and Terumo Medical Corporation; and has received a research grant from Asahi Intecc. Dr Lo has received speaking and proctoring honoraria from Abiomed, Bio-Excel, Boston Scientific, Abbott, and Terumo; and has served as a member of the advisory board for Medtronic, Abbott, and Edwards Lifesciences. Dr Lim has received educational grants for conferences from Kaneka, Asahi Intecc, Alvimedica, Elixir, OrbusNeich, Medtronic, Boston Scientific, Biosensor, and Abbott Vascular; and has served on the Board of Directors for APCTO Club Ltd, which receives research funding from Abiomed, OrbusNeich, and Asahi Intecc. Dr Kao has received honoraria for travel and accommodation from Abbott Vascular, Abiomed, Asahi Intecc, B Braun, Biotronik, Boston Scientific, Kaneka, Medtronic, Microport, and Terumo; has received a research grant from Elixir; and is a consultant for Boston Scientific, Asahi Intecc, and Kaneka. All other authors have reported that they have no relationships relevant to the contents of this paper to disclose.
